# Toward Quantification of Agitation in People With Dementia Using Multimodal Sensing

**DOI:** 10.1093/geroni/igac064

**Published:** 2022-10-15

**Authors:** Hannah Davidoff, Laura Van den Bulcke, Mathieu Vandenbulcke, Maarten De Vos, Jan Van den Stock, Nick Van Helleputte, Chris Van Hoof, Maarten J A Van Den Bossche

**Affiliations:** Department of Electrical Engineering (ESAT), KU Leuven, Heverlee, Belgium; CSH (Circuits and Systems for Health) - imec, Heverlee, Belgium; Department of Geriatric Psychiatry, University Psychiatric Center KU Leuven, Leuven, Belgium; Center for Neuropsychiatry, Research Group Psychiatry, Department of Neurosciences, Leuven Brain Institute, KU Leuven, Leuven, Belgium; Department of Geriatric Psychiatry, University Psychiatric Center KU Leuven, Leuven, Belgium; Center for Neuropsychiatry, Research Group Psychiatry, Department of Neurosciences, Leuven Brain Institute, KU Leuven, Leuven, Belgium; Department of Electrical Engineering (ESAT), KU Leuven, Heverlee, Belgium; Department of Development and Regeneration, Faculty of Medicine, KU Leuven, Leuven, Belgium; Center for Neuropsychiatry, Research Group Psychiatry, Department of Neurosciences, Leuven Brain Institute, KU Leuven, Leuven, Belgium; CSH (Circuits and Systems for Health) - imec, Heverlee, Belgium; Department of Electrical Engineering (ESAT), KU Leuven, Heverlee, Belgium; imec OnePlanet, Wageningen, Netherlands; Department of Geriatric Psychiatry, University Psychiatric Center KU Leuven, Leuven, Belgium; Center for Neuropsychiatry, Research Group Psychiatry, Department of Neurosciences, Leuven Brain Institute, KU Leuven, Leuven, Belgium

**Keywords:** Agitation in dementia, Multimodal sensing, Wearable sensors

## Abstract

**Background and Objectives:**

Agitation, a critical behavioral and psychological symptom in dementia, has a profound impact on a patients’ quality of life as well as their caregivers’. Autonomous and objective characterization of agitation with multimodal systems has the potential to capture key patient responses or agitation triggers.

**Research Design and Methods:**

In this article, we describe our multimodal system design that encompasses contextual parameters, physiological parameters, and psychological parameters. This design is the first to include all three of these facets in an *n* > 1 study. Using a combination of fixed and wearable sensors and a custom-made app for psychological annotation, we aim to identify physiological markers and contextual triggers of agitation.

**Results:**

A discussion of both the clinical as well as the technical implementation of the to-date data collection protocol is presented, as well as initial insights into pilot study data collection.

**Discussion and Implications:**

The ongoing data collection moves us toward improved agitation quantification and subsequent prediction, eventually enabling just-in-time intervention.

**Translational Significance:** Agitation is very often the most debilitating symptom of dementia, leading to institutionalization ahead of cognitive decline. Currently, treatment of agitation is based solely on partial and subjective observations from caregivers on what a person with dementia is experiencing. With the method presented here, we aim to quantify agitation in an objective, direct, and automated way. The understanding of agitation, increased by the output of this study, can be used to decrease agitation occurrence through timely intervention, monitor pharmacological and nonpharmacological interventions more effectively, increase the quality of life for both patients and caregivers, and reduce economic costs.

## Background and Objectives

As age is the leading risk factor for most forms of dementia, an aging population worldwide means an increase in the number of persons with dementia. Globally, there are currently over 50 million persons with dementia, a number expected to reach 82 million in 2030 and triple to 152 million in 2050 (Dementia Statistics | Alzheimer’s Disease International (ADI), n.d.). Dementia is characterized by a loss of cognitive functioning (memory, language, judgment, etc.). However, the behavioral and psychological symptoms of dementia (BPSD), such as apathy, depression, anxiety, sleep disturbances, psychotic symptoms, and agitation, are as clinically relevant as their cognitive counterparts (Steinberg et al., 2008).

One of the most debilitating BPSDs, agitation, of which a consensus definition was reached in 2015 by the International Psychogeriatric Association (Cummings et al., 2015), encompasses various behaviors like verbal and physical aggression, and excessive motor activity (Cohen-Mansfield & Billig, 1986). BPSDs, like agitation, are associated with faster cognitive decline and progression of dementia (Canevelli et al., 2013; Ismail et al., 2016), lower quality of life of patients (Karttunen et al., 2011; Tatsumi et al., 2009), a substantial increase in caregiver burden (Chen et al., 2018; Ryu et al., 2011) and higher economic costs (Costa et al., 2018). Although common, the fundamental physiology marking the occurrence of agitation, or what causes agitation, has not yet been concretely defined. The management of agitation by nonpharmacological and pharmacological interventions is thus often solely based on subjective assumptions by caregivers about what the person with dementia is experiencing. Treatment of agitation is mainly focused on intervention when symptoms occur, as opposed to more preventative measures. Given the disruptive nature of agitated behavior and the limited understanding of underlying causes of agitation, agitation can lead to the (over)use of psychotropic medications like antipsychotics and benzodiazepines, even though these medications lead to severely increased morbidity and mortality in older adults and especially in persons with dementia (Salzman et al., 2008; Selbæk et al., 2008; Spek et al., 2016; Wetzels et al., 2011). Therefore, there is a distinct need for direct and objective monitoring of agitation. We aim to build the foundation to enable this need through our multimodal sensor system; combining measures of the living environment of people with dementia as well as physiological data from wearables to uncover physiological markers and quantify possible triggers of agitation.

Current research has started to quantify the living environment of persons with dementia, aiming to uncover triggers of symptoms like agitation. Several theoretical models of the cause of agitation, such as Cohen-Mansfield’s “Unmet Needs” model (2015), the Progressively Lowered Stress Threshold model (Gerdner et al., 2005; Hall & Buckwalter, 1987; Richards & Beck, 2004; Smith et al., 2006), and the Environmental Docility (Lawton & Simon, 1968) hypothesis attribute these symptoms to a complex mechanism, taking into account the patient’s environment as well as internal thoughts or feelings that persons with dementia can often no longer express due to disease progression. These models provide the foundation for the clinical interpretation of agitation and are the basis of subsequent study designs. Several studies show the correlation of single environmental parameters, such as temperature (Tartarini et al., 2017), sound (Janus et al., 2020; Joosse, 2012), light (Figueiro, 2017), and humidity (Au-Yeung et al., 2020), to the level of agitation in a patient group. While these studies are useful in learning about the possible triggers of agitation, they lack the ability to characterize the patient’s environment as a whole. Sensory under- or overstimulation of persons with dementia is hypothesized to be one of the main causes of an agitation episode. However, overall environmental stimuli do not only arise from a single environmental parameter; to quantify stimulation, all environmental parameters (e.g., temperature, light, humidity, and sound) must be considered together. More recent studies aimed to take this into account by utilizing a multimodal sensing setup. A multiphase study called “Behavioral and Environmental Sensing and Intervention” (BESI) combined environmental parameters, patient movement with an actigraphy wearable, and annotation of agitation events (Bankole et al., 2020). A Toronto-based group developed and used their multimodal sensing system, Detect Agitation and Aggression in Dementia (DAAD), in a 17-patient pilot study (Spasojevic et al., 2021). DAAD combines movement and physiological parameters from a patient-worn wearable, with agitation annotation from direct observation with timestamps later refined by retrospective analysis of video (Spasojevic et al., 2021). A third study follows a similar setup to the BESI study, but also includes physiological parameters measured during sleep (Au-Yeung et al., 2020).

However, in the aforementioned studies, there is always at least one facet of the necessary multimodal design lacking. Either studies focus on physiological parameters without contextual parameters, or they focus on contextual parameters, but lack physiological parameters, particularly during the day. The inclusion of physiological parameters in a multimodal sensor system design, like the one described in this article, is invaluable in this patient population (Choi et al., 2022). These parameters often correlate with mental states and can be used as a form of unspoken communication taking the place of the hampered ability of persons with dementia to communicate their internal feelings or needs. The correlation of physiological parameters, like those measured by wearable sensors, to agitation can therefore lead to more objective monitoring of agitated behavior in persons with dementia. For example, a sensor measuring electrodermal activity (EDA) can be used in conjunction with other data to quantify a person’s autonomic nervous system (ANS) activation and subsequently characterize a person’s arousal state (Melander et al., 2017, 2018; Nath et al., 2022). It is hypothesized, in models such as the Progressively Lowered Stress Threshold (PLST) model (Hall & Buckwalter, 1987), that agitation is closely related to stress. Therefore, it is reasonable that measuring ANS activation with sensor signals, typically used to quantify stress, can also give us an idea of a patient’s level of agitation, or susceptibility to future agitation.

Given the theoretical models of agitation and knowledge from existing single-parameter research, the gap in the understanding of agitation likely stems from a lack of all-encompassing data in current research. Due to its multifaceted design, the multimodal sensor system detailed in this article expands the possibilities of the outcome of earlier observational research from limited, parameter-specific, observations to more comprehensive ones. This system allows not only for the modeling of individual parameters and their relationship with agitation, but also of the interaction between parameters and their combined effect on agitation. The external contextual and internal physiological parameters will be assessed in association with psychometric scales filled in real-time by clinical staff quantifying the subjective experience of agitation. This lays the groundwork for using real-time physiological data to understand psychological state without direct observation by a trained professional. The study protocol described in this article is designed to, once we have collected all the data, answer several main research questions relating to the cause and markers of agitation.

(1) What are the physiological markers of agitation?
(2) What triggers agitation episodes? Are triggers consistent over the population or are they individual?

The method of data collection in this study, including insight into pilot data, will be presented in this article along with a discussion with both clinical and technical perspectives on the study design and plans for future analysis.

## Research Design and Methods

### Study Population

The protocol of this study (approved by the Ethics Committee Research of University Hospitals Leuven with ID: S62882) is integrated in the day-to-day work at a specialized neuropsychiatric ward within a psychiatric hospital, UPC KU Leuven. This specialized ward, called Cog K, focuses on persons with dementia with severe behavioral problems. The population of patients admitted to the ward consists of people classified within Tier 6 and Tier 7 of Brodaty, Draper, and Low’s seven-tiered model of BPSD care: “Dementia with very severe BPSD” and “Dementia with extreme BPSD,” respectively (Brodaty et al., 2003). The prevalence of persons with dementia classified into these tiers is estimated at ~1% of the broader population of people with dementia (Brodaty et al., 2003).

#### Patient intake

Patients at the ward can be included if they have a Diagnostic and Statistical Manual of Mental Disorders-5 diagnosis of a major cognitive disorder (e.g., dementia). Informed consent will mainly be obtained through the patient’s representative. However, information on the study and what is expected of the patient will always be explained as much as possible, and in a way corresponding to the patient’s current cognitive capacities. After consent is obtained, three psychometric scales are completed: the Cohen-Mansfield Agitation Inventory, the Neuropsychiatric Inventory, and the Cornell Scale for Depression in Dementia. Additionally, sex, age, subtype of dementia, duration of dementia, career, and current medication are also recorded. The degree of independent mobility of the patient (e.g., confined to wheelchair, aided or unaided independent movement), as well as any comorbid psychopathologies are also noted and will thus be considered throughout data analysis, however, these are not exclusion criteria. Exclusion criteria related to the type of dementia will not be used, in order to form a patient sample representative of the overall population of persons with dementia showing agitation.

### Agitation Annotation

**Figure 1.**
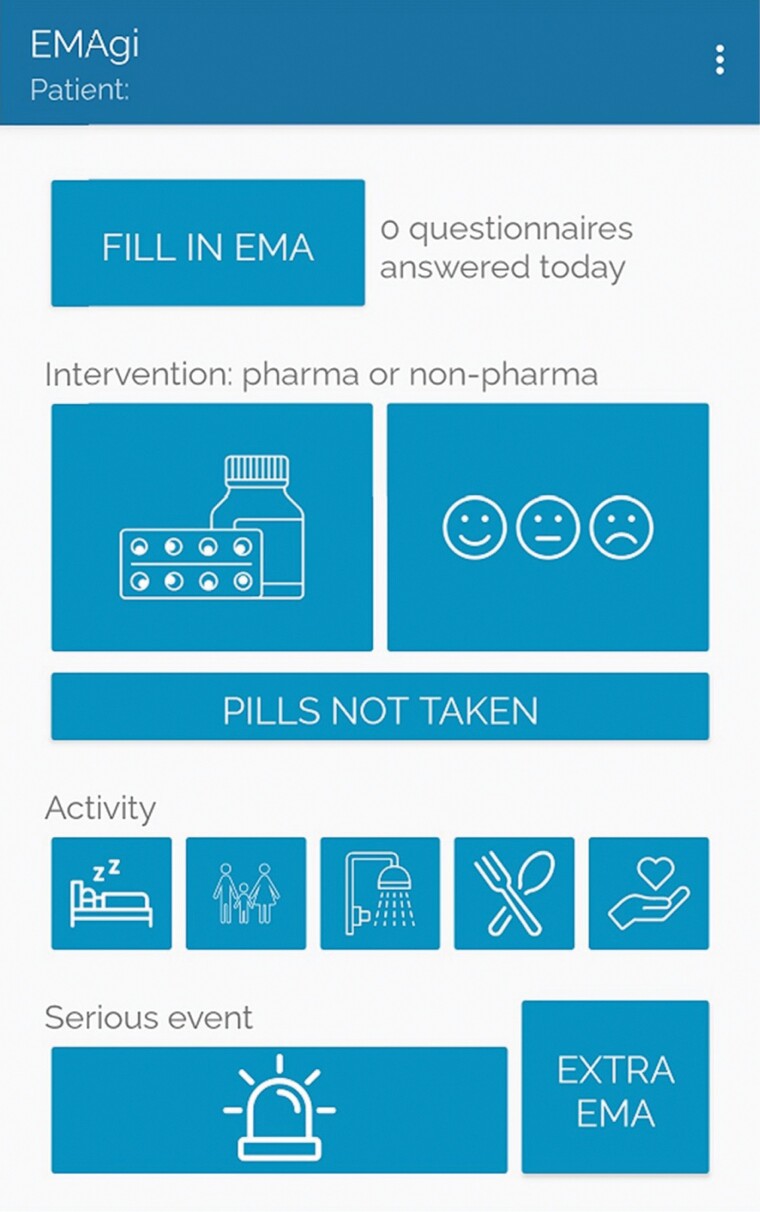
EMAgi app developed by the EDiT team of the Interuniversity Micro-Electronics Center (imec). The contextual annotation buttons seen here (by row from left to right). Row 1: pharmacological or nonpharmacological intervention, medication refused “Pills not taken”; row 2: activities—sleep, visit, bathing, eating, and care; row 3: serious event and button for extra EMA.

The nurses at the ward are responsible for the agitation annotation (prompted by signal) of the data set as well as the annotation of activities throughout the day (unprompted). Annotation is facilitated through a dedicated study phone (Nokia 6.1 and Android OS) with a specialized and study-specific app (Figure 1). The contextual annotations possible are shown on the app screen in Figure 1. This app, EMAgi, integrates the Experience Sampling Methodology (ESM), to capture the level of patient agitation at pseudo-random time intervals. It does this by notifying the nurse carrying the study phone nine times per day, between the hours of 0845 and 2045. This notification can be clicked to open the agitation survey: the Pittsburgh Agitation Scale (PAS) and Richmond Agitation Sedation Scale (RASS). After seeking out and observing the patient at the time of survey notification, the survey is filled in by one of the nurses directly responsible for the care of patients on the ward (31). The PAS encompasses four subscales (one per agitation subtype): motor agitation, verbal agitation, aggression, and resistance to care (Rosen et al., 1995). Each of these subscales is scored from 0 (not present) to 4. The PAS has been found to be reliable and easy to use in a study by Rosen et al. (1995) with total score correlations of 0.82 when compared between four nurses on an inpatient geriatric unit. After completion of the PAS, the nurses fill in the RASS (Sessler et al., 2002). This is a one-question numeric scale ranging from −5 (unarousable) to +4 (combative), with an interrater validity of κ = 0.91 (Sessler et al., 2002). It is also possible for the nurses to fill in a survey when they observe agitation through the “Extra Ecological Momentary Assessment (EMA)” button in the app. After each patient inclusion, we record the occurrence and timestamp of aggression incidents noted in the clinical file, if applicable.

### Technical Implementation

**Figure 2.**
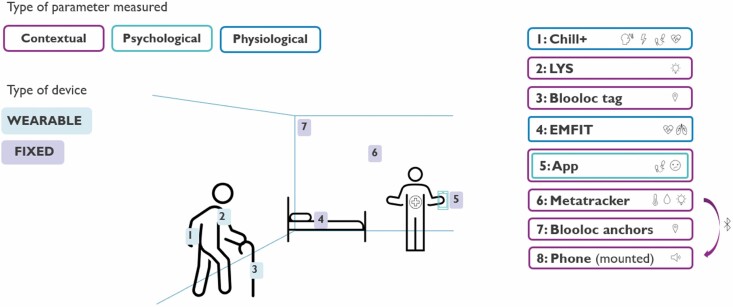
Complete multimodal system design. Three wearables: Chill+ (1), LYS (2), and the Blooloc tracker (3). Nighttime physiological sensor: EMFIT (4). The specialized app (5) made for the study. And fixed sensors: the Metatracker (6) for environmental parameter measurement, Blooloc anchors (7) that localize the tracker (3), and the Nokia phone (8) that collects and packages data from the Metatracker and its own microphone. This implementation is explained more in-depth in the “Technical Implementation” section.

To quantify a patient’s living environment and agitation state in the most complete manner, we devised a multimodal sensor system to measure psychological, contextual, and physiological parameters (synchronized by timestamp). The system consists of fixed sensors installed at the ward, wearable sensors worn by the patient, and an Android phone outfitted with a mobile app developed specifically for the data collection in this study. An overview of the system design can be seen in Figure 2. The study protocol is designed to be integrated into the ward protocols with minimal impact on standard procedures.

#### Fixed sensors

The fixed sensors measure the environmental parameters on the ward, the patient location, and the nighttime physiological parameters of a patient during sleep.

To characterize the environment, 11 sensor enclosures were installed in the ward. These sensor enclosures cover the living and dining rooms, hallways, bathrooms, and the included patient room. Included in each enclosure were: the Metatracker from MbientLab and a Nokia 6.1 Android phone. The Metatracker has the capability to measure data in multiple modes; for the purpose of this study, three of the sensor modes were selected. Details of the sensor enclosure can be found in Table 1. This Metatracker streams data via BLE to the phone mounted in the same enclosure. The phone is equipped with an app (EnV app) tailor-made for this study to structure incoming data, package it and send the packaged data to the backend environment in the cloud. The incoming data can then be monitored remotely via a dashboard to ensure data completeness. This dashboard can also be used to verify live sensor status (e.g., sending data, battery levels, and charging status). In the event of a connectivity problem, the phone acts as a temporary storage solution.

**Table 1.** Data Streams Collected by Sensor Enclosure, Grouped by Device Mounted in the Enclosure

Device	Data stream
Metatracker	Light intensity (lux)
	Relative humidity (%)
	Temperature (deg. C)
Nokia 6.1	A-weighted dB level (dB)
	dB in octave bands (dB, 1.32)

The microphone on the Nokia device measures, facilitated by the EnV app, decibel levels in octave band frequency ranges, as well as calculating the DB-Leq and A-weighted values. This data are sent with the environmental data measured by the Metatracker to the cloud-based backend.

In addition to the environmental sensors, 33 BLE devices from Blooloc (Blooloc YooBee Sensors, Trackers, and Hubs, n.d.) were installed at the ward to enable patient-localization. These include 30 BLE solar-powered anchors, and 3 BLE-enabled hubs with Ethernet connection. The tracker wearable, also BLE enabled, communicates with each of the anchors and/or hubs when the patient is present in the ward. The patient’s x–y coordinates and their “uncertainty” are determined based on the relative signal strength from the tracker to each of the nearby anchors or hubs. With this BLE installation, we can localize the patient on the ward with an accuracy of 1–2 meters.

To characterize the patient’s physiological parameters during sleep, we use the EMFIT QS ballistocardiography-based under-mattress sensor (manufactured by EMFIT, Finland). This sensor has the capability to measure fine movements through piezoelectric elements. Using these measurements in different frequency bands 0.07–3 Hz (fs = 25 Hz, for respiration) and 1–35 Hz (fs = 100Hz, for heart rate [HR]) the sensor, and companion backend with integrated algorithms, can extract features characterizing sleep physiology, including HR, respiration rate, heart rate variability (HRV), movement, HRV-based ANS recovery, as well as estimating sleep time, and sleep classes.

#### Wearables

Three wearables are included as a part of the sensor system: the Chill+, the Blooloc tracker, and the LYS button sensor. Each of these wearables covers a different sensor modality.

The imec-developed Chill+ is a wrist-worn physiological and actigraphy wearable that measures: EDA, HR via photoplethysmography (PPG), movement via an accelerometer and gyroscope, and skin temperature. These measures are recorded at a sampling frequency of 256, 256, 32, and 1 Hz, respectively. The Blooloc tracker, in the form of a watch-like wristband, works together with the fixed BLE installation and can be worn on the opposite wrist of the Chill+, attached to a belt-loop, or placed in a pocket. The third wearable is the LYS light button sensor from LYS Technologies. This 4g sensor is clipped on the outermost layer of the patient’s clothing at breast level or higher to measure the light closest to the eyes. The light measured from a point on the body, closer to the eyes, is more representative of the light affecting the patient than that measured by a wrist-worn light sensor. Due to the low weight of this sensor, the light measurement causes little to no disturbance to the patient. The LYS sensor measures light intensity in lux, melanopic lux, red, green, blue (RGB) and infrared (IR) values, color temperature in Kelvin and movement, once per 15 s. Including this sensor in a multimodal sensing system allows for the characterization of patient-specific light exposure as opposed to the more general environmental light values. This is the first multimodal sensing study to integrate this type of patient-specific measurement of environmental parameters.

### Preliminary Data Pipeline

**Figure 3.**
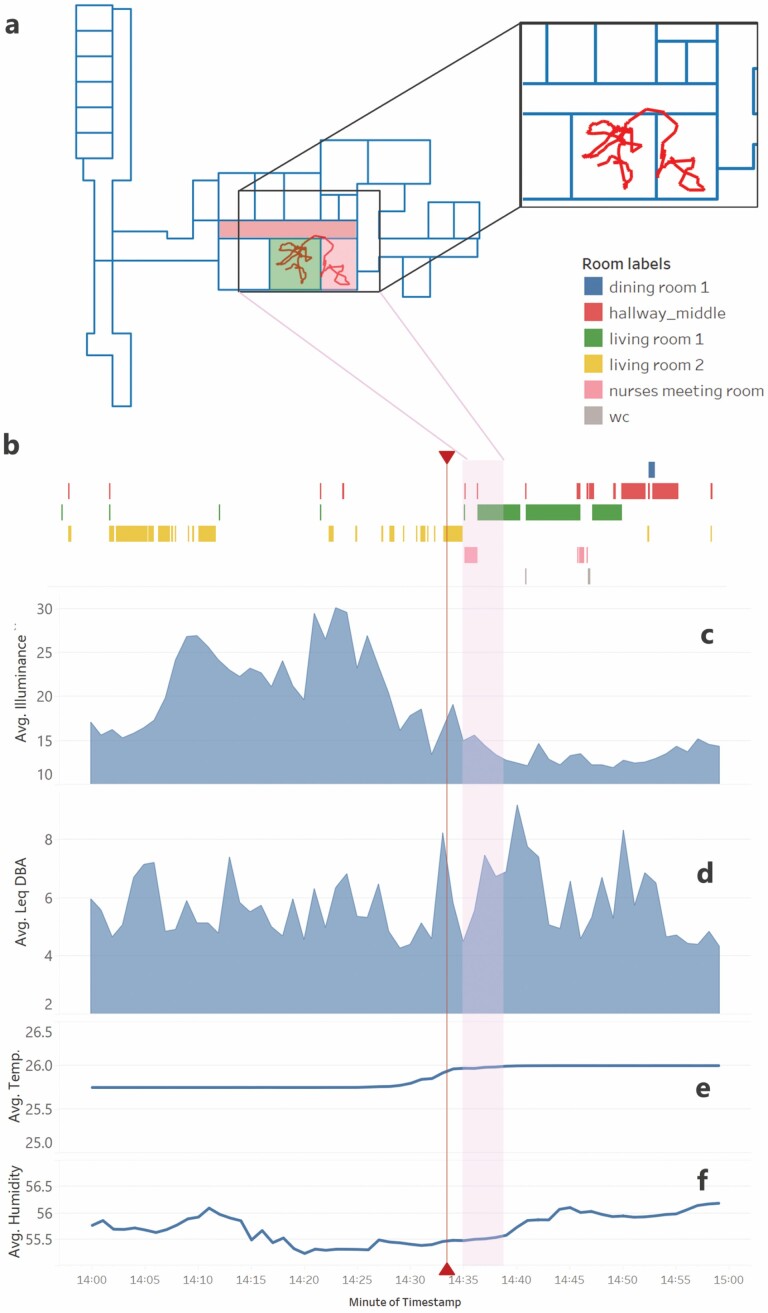
One hour of multimodal data; starting from the raw Blooloc trajectory (A), to the processed room labels (B), and four environmental data streams collected from the “living room 1” sensor enclosure location: illuminance (in lux), (C), A-weighted DB value (D). Temperature in degrees Celsius (E) and the relative humidity (F). The average for each of the four plots (C–F) is the average over each minute. Marked by a vertical line bookended by two triangles is the timestamp of agitation annotation (14:33). At this moment in time, the patient scored two on the verbal agitation subscale of the PAS, leading to a total score of 2. The raw trajectory shown in (A) is a 3-min segment from 14:35:01 to 14:38:27. This timeframe is marked with the shaded area across plots (C-F). The background of (A) is the ward floor plan. The relevant rooms for this trajectory are color-coded in correspondence with the colors of the legend, and room labels (B). The trajectory depicted, as well as the corresponding environmental data, serves to give insight into a patient’s movement pattern (possibly indicative of motor agitation) as well as the external environmental factors that may be influencing a patient’s agitation state. PAS = Pittsburgh Agitation Scale.

The raw location data from the Blooloc system is fed into a processing pipeline which adjusts any data points that fall outside of the defined ward area, as well as impossible room transitions, and outputs adjusted x–y coordinates, accuracy of the position, and room-level labels. Using the room label output, we can analyze environmental data of the nearest sensor enclosure in that time window. The location data shown in Figure 3A and B provides the basis for subsequent analysis of environmental parameters (Figure 3C–F) related to agitation, as well as standing alone in characterizing patient movement that may be indicative of motor agitation.

Figure 3 serves to provide initial insight into how the data from multiple sensor modalities can be combined. Analysis of this multimodal data and synchronized physiological and contextual data from the wearables (Chill+ and LYS) will start with feature extraction of features from literature, in addition to combined features. Feature selection techniques will be used to reduce the dimensionality of the input into the subsequent models/classifiers used. This research aims to design a classifier for agitation based on the resulting reduced feature sets. Given the expected sparse nature of the agitation labels, we will likely use multiple instance modeling, anomaly detection models, or one-class Support Vector Machines to classify agitation instances.

The interpretation of the multimodal data is done on both a parameter-specific level and on a multivariate level, taking multiple parameters and their influence on each other into account simultaneously. The study design allows for both a supervised (including agitation annotation) and an unsupervised (not including agitation annotation) analysis of the data. Initial analysis of the data will use the agitation annotation as the ground truth and guide the analysis of the other time series data. Combining both the theoretical models of the cause of agitation (Cohen-Mansfield & Billig, 1986; Hall & Buckwalter, 1987; Lawton & Simon, 1968) and the existing literature on univariate correlations with agitation, we can analyze the data in various length time windows before annotation. This aims to form a clearer picture of the impact of the living environment of persons with dementia on their level of agitation. Due to the continuity of the data collection protocol and considering the total inclusion length per patient, this can be done on timescales ranging from a minute to a day.

## Discussion and Implications

With the goals of determining the physiological markers of agitation and identifying corresponding triggers, we developed a holistic, multimodal sensing system.

### Clinical Discussion

#### Agitation annotation

Using the ESM to quantify agitation in people with dementia is a novel application of this method. Related research (in neurodegenerative disease) has used this method to quantify a caregiver’s mental state and experiences when involved in the life of a person with dementia (Knippenberg et al., 2018), as well as to assess depression in people with dementia (Niculescu et al., 2021), but not yet agitation. Existing multimodal studies on agitation have labeled the data either through direct observation (Homdee et al., 2019), or through retrospective analysis based on the available video to refine timestamps of prospective annotation (Spasojevic et al., 2021). Both methods only label agitation incidents when they happen but not the absence of agitation. The frequency and content of the ESM, as it is used in this protocol, allows for a more detailed characterization (e.g., presence/absence of agitation subtypes through PAS subscales) of the behavior of persons with dementia than the typical binary characterization of the presence or absence of agitation. This subsequently enables modeling of different subtypes of agitation and investigation into subtype-specific triggers. The frequency of the agitation annotation throughout the day enables investigation into the progression of the amount of agitation, as well as looking more specifically at which subtype(s) of agitation is/are seen at the moment of observation. Initial pilot data on survey completion rates and agitation prevalence for these patients suggests that using the ESM in this protocol is feasible and captures agitation events. With the knowledge of agitation gained in this study, we can eventually model individual agitation responses without reliance on frequent data annotation by care staff. This more objective modeling of agitation responses could lead to more adequate prescribing of psychopharmacological treatments, which is especially relevant as older adults with dementia are very prone to serious adverse events associated with the use of, for example, antipsychotics (Salzman et al., 2008). It could also lead to a decrease in caregiver burden, allowing older adults to stay at home for a longer time, and slowing down the need for institutionalization in nursing homes.

Although often used in the Intensive Care Unit, the RASS was included in the ESM survey because of its ease-of-use, time to complete, and interrater validity (Sessler et al., 2002). It gives a quick numerical insight into the severity of agitation at that moment. When used in conjunction with the PAS, we can verify that both scores are representative of the patient’s psychological state. Because of the interrater validity, the rotating shifts of nurses at the ward should not negatively influence the quality of the agitation annotation.

#### Interpretation of data and study design

When some patients are most agitated, they take the wearables off. This can lead to crucial gaps in data which may be interpreted as agitation itself. In this patient population, explaining the study to the patient, for example, why they are wearing the wearables, can help in patient compliance. Other times, the patient is not acutely aware of the wearables leading to higher compliance. During initial patient inclusion in the pilot, we have noticed a few study limitations. As not all rooms were outfitted with the study sensors, this sometimes meant that patients had to be moved between rooms for study participation. From a clinical perspective, this is to be avoided because of the increased risk of disorientation. Additionally, the degree of independent mobility of a patient is considered when analyzing movement and location data, as this can affect the subsequent clinical interpretation.

Regarding study design, we do not include parameters that capture the interaction between specific staff and the patient, nor the interactions between patients that could have an impact on a patient’s state of agitation. This was not integrated because of the magnitude of complexity this would add to the overall project.

### Technical Discussion

In addition to clinical integration difficulties, technical challenges were also encountered. The agitation annotation was indicated by notifications on the study phone. However, due to connectivity issues at the ward (Wi-Fi or cell service instability) occasional notifications were not sent to the study phone, leading to missing agitation labels. The impact of this was mitigated using a dedicated sim card to ensure cell service subscription as a backup for the Wi-Fi as well as monitoring the number of surveys filled in throughout the day. Another point to consider was the volume of the study phone, as during busy moments at the ward, survey notifications would sometimes be missed due to not being loud enough to be noticed. Other challenges include the occasional overlooking of a step of the protocol where the LYS data needed to be synchronized with the study iPhone, leading to partial data loss. Finally, all incoming data must be integrated and synchronized (in the time-domain) for the analysis to occur on a multivariate level. This integration can be an intense workload and is ongoing to streamline the process for new incoming data.

Despite the challenges, the pilot study has shown the feasibility of integrating a multimodal sensing protocol into a clinical day-to-day environment; and the resulting data shows that we can quantify and define the living environment of a person with dementia in an all-encompassing manner.

## Conclusion and Future Work

The increased knowledge derived from this study protocol has the potential to provide the foundation for new technological developments or intervention systems that can lessen the negative impact of agitation on the quality of life of persons with dementia and their caregivers. The ability to detect agitation markers enables more accurate and objective monitoring of patients with dementia in both clinical care and study settings. This objective marker can also be used to optimize both pharmacological and nonpharmacological interventions, subsequently improving care and quality of life. Additionally, the detection of agitation can facilitate prediction, which can lead to prompted just-in-time interventions, eventually decreasing agitation occurrence. Moving toward the prediction of agitation can be done by combining the knowledge gained through this protocol of both physiological markers and contextual triggers of agitation. When integrated, the foundational output of this study can result in decreased caregiver burden, more effective tailored treatment plans, and increased quality of life for both patients and caregivers.
